# Disaster preparedness perceptions and associated factors among final-year medical students in Hatay and Ordu, Türkiye

**DOI:** 10.1371/journal.pone.0358641

**Published:** 2026-10-06

**Authors:** Yusuf Demirtaş, Saime Şahinöz

**Affiliations:** Department of Public Health, Faculty of Medicine, Ordu University, Ordu, Türkiye; University of Luzon, PHILIPPINES

## Abstract

**Introduction:**

Following the February 2023 Türkiye earthquakes, disaster preparedness among healthcare professionals and medical students has become an important public health concern, particularly in disaster-prone settings where newly graduated physicians may be rapidly deployed to frontline healthcare services. However, evidence on disaster preparedness perceptions among medical students remains limited, especially in comparative settings involving regions with different levels of disaster exposure. This study aimed to assess disaster preparedness perception and associated factors among final-year medical students studying in two provinces of Türkiye with differing disaster risk profiles in the aftermath of the February 2023 earthquakes.

**Methods:**

A cross-sectional study was conducted between April and May 2023 among final-year medical students from Hatay Mustafa Kemal University and Ordu University, Türkiye. Data were collected through a structured self-administered online questionnaire covering sociodemographic and disaster-related characteristics, an author-developed earthquake healthcare knowledge test, and the Disaster Preparedness Perception Scale of Personnel Working in Pre-Hospital Emergency Health Services (HASPAHA). Univariable analyses and multivariable logistic regression were performed to identify factors independently associated with high perceived preparedness, which was defined using a median split.

**Results:**

A total of 188 students participated (109 in Hatay, 79 in Ordu). The mean disaster preparedness perception score was 101.5 ± 13.6 (scale range: 28–140), with significantly higher scores observed in Hatay compared to Ordu. In multivariable analysis, male sex (aOR=3.13) and studying in Hatay (aOR=2.11) were associated with high perceived preparedness. Willingness to volunteer in disaster healthcare services (aOR=3.99) and higher earthquake healthcare knowledge (aOR=2.95) were also significant associated factors.

**Conclusions:**

Among final-year medical students, perceived preparedness was associated with regional disaster exposure context and motivational characteristics, alongside earthquake healthcare knowledge. To strengthen future physicians’ preparedness, medical curricula could incorporate experiential disaster training, opportunities for voluntary participation in disaster response, and structured disaster preparedness education.

## Introduction

The devastating February 2023 earthquakes in Türkiye underscored the extensive clinical and organizational responsibilities faced by physicians in large-scale disaster settings, while also highlighting the increasing importance of disaster preparedness [1,2]. In countries with high disaster risk, ensuring that future physicians possess adequate preparedness and response capacity is vital to mitigating disaster-related health consequences, since newly graduated physicians are frequently deployed in frontline healthcare services during such catastrophic events [3].

Türkiye is among the most disaster-prone countries in the world due to its tectonic, seismic, topographic, and climatic characteristics [4]. Among these hazards, seismic risk is predominant, with earthquakes accounting for more than 90% of disaster-related fatalities [5]. Two major earthquakes with magnitudes of 7.7 and 7.6 were recorded in southeastern Türkiye on February 6, 2023, occurring approximately nine hours apart. Affecting 11 provinces, the earthquake sequence resulted in substantial loss of life and widespread structural damage. In terms of mortality, this event represents the deadliest earthquake disaster in Türkiye’s history [6]. Hatay province, located in southern Türkiye, is recognized as a region with a high seismic risk. It was the most severely affected province during the February 2023 twin earthquakes, with more than 21,000 deaths estimated to have occurred in Hatay alone [7,8]. Türkiye encompasses a wide and geographically diverse territory, resulting in substantial regional variation in disaster risk across provinces. In contrast to Hatay, Ordu province, located in northern Türkiye, is characterized by a comparatively lower seismic risk, was not directly affected by the February 2023 earthquakes, and has not experienced disaster-related mortality on a comparable scale in recent years [9].

Studies conducted across different countries have reported that, despite their critical roles in disaster settings, healthcare workers and healthcare students often exhibit low levels of perceived disaster preparedness, alongside reported limitations in confidence and comfort in performing disaster-related tasks [10–12]. Disaster preparedness perceptions may be influenced by multiple factors, including demographic characteristics, disaster-related knowledge, and intentions to volunteer in disaster healthcare services. Furthermore, regional disaster risk and prior disaster experience may further shape preparedness perceptions [13–15]. Particularly in countries such as Türkiye, which face substantial disaster risk, it is important to comprehensively evaluate disaster preparedness perceptions and multiple potentially associated factors among medical students who may be involved in disaster response. However, evidence from Türkiye in this population remains limited, particularly regarding comparisons between regions with markedly different levels of disaster exposure and risk. To our knowledge, no previous study has compared disaster preparedness perceptions across provinces with differing disaster risk profiles.

Therefore, this study aimed to assess disaster preparedness perceptions and associated factors among final-year medical students at Hatay Mustafa Kemal University and Ordu University, Türkiye, following the February 2023 earthquakes.

## Materials and methods

### Study design and setting

This cross-sectional study was conducted between 20 April and 31 May 2023 among final-year (sixth-year) medical students from two universities in Türkiye. The study included students from Hatay Mustafa Kemal University Tayfur Ata Sökmen Faculty of Medicine, located in Hatay Province in southern Türkiye — the region most severely affected by the February 2023 earthquakes with the highest numbers of deaths and injuries — and from Ordu University Faculty of Medicine, located in Ordu Province in the northern part of the country, a region with lower disaster risk and without any recent large-scale disasters comparable to those in Hatay. A census-based recruitment approach with voluntary participation was used. All eligible final-year medical students at both faculties were invited to participate. Prior to participant recruitment, twenty students from Hatay were intentionally not contacted due to earthquake-related bereavement or psychological vulnerability, in accordance with ethical considerations. A total of 79 out of 85 final-year students from the Faculty of Medicine, Ordu University, participated in the study (response rate: 92.9%). At Hatay Mustafa Kemal University Tayfur Ata Sökmen Faculty of Medicine, 161 of the 181 final-year students were invited to participate, and 109 students completed the survey (response rate: 67.7%). The overall response rate of the study was 76.4%.

### Data collection tool

Data were collected through a structured online questionnaire. The questionnaire consisted of three sections. The first section included questions on participants’ age, sex, previous participation in disaster-related health responses, and willingness to participate in disaster healthcare services. The second section comprised an earthquake healthcare knowledge test developed by the research team directly from the national guide on health services in disasters published by the Turkish Medical Association [16]. Item development was guided by the core topics addressed in the guideline, and the final test consisted of 10 multiple-choice questions covering key domains of earthquake-related healthcare (e.g., triage, medical management during rescue, crush syndrome, post-earthquake infectious diseases, vaccination, environmental health, and mental health). The items were reviewed by two external experts in disaster medicine and public health for content relevance, clarity, and appropriateness in relation to disaster healthcare competencies. Items were revised iteratively based on their feedback until consensus was reached. In addition, pilot testing was conducted among a small group of students prior to the main study to assess clarity and comprehensibility of the items. However, no formal psychometric validation was conducted prior to use. Each correct answer was scored as 1 point and each incorrect answer as 0, yielding a total possible score ranging from 0 to 10, with higher scores indicating greater knowledge. The third section included the Disaster Preparedness Perception Scale of Personnel Working in Pre-Hospital Emergency Health Services (HASPAHA), developed by Tercan and Şahinöz [17]. The scale consists of 28 items rated on a 5-point Likert scale (1 = strongly disagree to 5 = strongly agree), with total scores ranging from 28 to 140. Higher scores indicate a higher perception of disaster preparedness. The original validation study reported a Cronbach’s alpha coefficient of 0.925 for the scale. The Cronbach’s alpha coefficient for the scale was 0.905 in the present study.

### Statistical analysis

Descriptive statistics were presented as frequencies and percentages for categorical variables, and as means and standard deviations or medians and interquartile ranges for continuous variables. The normality of continuous variables was assessed using the Kolmogorov–Smirnov and Shapiro–Wilk tests, supported by visual inspection of histograms and Q–Q plots. As the data were not normally distributed, non-parametric tests were used for comparisons. Comparisons of continuous variables between two groups were performed using the Mann–Whitney U test. Associations between categorical variables were examined using the Pearson’s chi-square test. Both the earthquake healthcare knowledge test and disaster preparedness perception scores were dichotomized based on their median values to facilitate categorical comparisons and logistic regression analyses, given the exploratory nature of the study and the absence of established validated cut-off points for these measures in the study population. Univariable logistic regression analyses were performed to estimate crude odds ratios (cORs), 95% confidence intervals (CIs), and p-values for the associations between each examined variable and high disaster preparedness perception. All six variables examined in the study were considered theoretically relevant based on the literature and were therefore included in the multivariable logistic regression model. All six variables were also significantly associated with disaster preparedness perception level in univariable analyses. The variables were simultaneously entered into the multivariable model using the enter method. Adjusted odds ratios (aORs) and 95% confidence intervals (CIs) were reported for the final model. Model fit was assessed using the Hosmer–Lemeshow goodness-of-fit test and Nagelkerke R². Multicollinearity among independent variables was assessed using tolerance and variance inflation factor (VIF) values. Tolerance values >0.20 and VIF values <5 were considered to indicate the absence of problematic multicollinearity. Potential interaction effects between university location and earthquake healthcare knowledge, and between university location and willingness to volunteer, were examined by including the corresponding interaction terms in separate multivariable logistic regression models. To evaluate the robustness of the findings and address potential limitations related to dichotomization, a supplementary sensitivity analysis using multiple linear regression was performed, in which disaster preparedness perception score, earthquake healthcare knowledge score, and age were analysed as continuous variables. Assumptions of linear regression were evaluated through inspection of residual plots and multicollinearity statistics. Unstandardized regression coefficients (B), standardized coefficients (β), and 95% confidence intervals were reported. There were no missing data for any of the variables included in the analyses. All statistical analyses were performed using IBM SPSS Statistics 24.0. A p-value of <0.05 was considered statistically significant.

### Ethical considerations

This study was conducted in accordance with the principles outlined in the Declaration of Helsinki. Ethical approval was obtained from the Ordu University Clinical Research Ethics Committee (date: April 14, 2023; number: 2023/106). All participants were informed about the purpose and procedures of the study, and electronic informed written consent was obtained from all participants prior to data collection through the online survey system. Participants were informed that the questionnaire was anonymous, and no personally identifiable information was collected.

## Results

A total of 188 students participated in the study, including 109 (58.0%) from Hatay Mustafa Kemal University and 79 (42.0%) from Ordu University. The mean age of the participants was 24.7 ± 1.6 years, with students from Hatay Mustafa Kemal University being older than those from Ordu University (25.0 ± 1.7 vs. 24.3 ± 1.2 years, p < 0.001). The majority of students were male in both universities. Previous participation in disaster-related health responses was reported by 22.3% of the students, with a significantly higher proportion observed among students from Hatay Mustafa Kemal University compared with Ordu University (33.9% vs. 6.3%, p < 0.001). Regarding willingness to participate in disaster healthcare services, 60.6% stated that they would volunteer, while 39.4% indicated that they would participate only if assigned or were unwilling. Table 1 presents the sociodemographic characteristics, disaster-related experience, and willingness to participate in disaster healthcare services among final-year medical students in Hatay and Ordu.

**Table 1:** Sociodemographic characteristics, disaster-related experience and willingness among final-year medical students in Hatay and Ordu.

Variable	Total		Hatay		Ordu		*p* (Hatay vs Ordu)
	n	%	n	%	n	%	
**Number of participants**	188	100.0	109	58.0	79	42.0	–
**Age, mean ± SD**	24.7 ± 1.6		25.0 ± 1.7		24.3 ± 1.2		**<0.001** ^†^
**Age group**							
≤ 24	101	53.7	50	45.9	51	64.6	**0.011** ^‡^
≥ 25	87	46.3	59	54.1	28	35.4	
**Sex**							
Male	101	53.7	58	53.2	43	54.4	0.869^‡^
Female	87	46.3	51	46.8	36	45.6	
**Previous participation in disaster-related health response**							
Yes	42	22.3	37	33.9	5	6.3	**<0.001** ^‡^
No	146	77.7	72	66.1	74	93.7	
**Willingness to participate in disaster healthcare services**							
Voluntarily	114	60.6	71	65.1	43	54.4	0.138^‡^
Only if assigned / Unwilling	74	39.4	38	34.9	36	45.6	

SD, standard deviation

^†^ Mann–Whitney U test; ^‡^ Pearson’s chi-square test

The earthquake healthcare knowledge test scores were similar between students from Hatay Mustafa Kemal University and Ordu University (6.15 ± 1.36 vs. 6.23 ± 1.37, p = 0.793). The overall disaster preparedness perception scale score (scale range: 28–140) in the total sample was 101.5 ± 13.6 (median [Q1–Q3]: 101 [93–109]). Final-year medical students in Hatay had significantly higher scale scores compared with those in Ordu (103.6 ± 15.5, median [Q1–Q3]: 104 [94–112] vs. 98.7 ± 9.8, median [Q1–Q3]: 99 [93–103], p = 0.013). Table 2 presents the comparison of earthquake healthcare knowledge test and disaster preparedness perception scale scores among the participants.

**Table 2:** Knowledge test and disaster preparedness perception scale scores among final-year medical students in Hatay and Ordu.

Variable	Total	Hatay	Ordu	*p* (Hatay vs Ordu)
**Earthquake healthcare knowledge test score (0–10)**				
Mean ± SD	6.18 ± 1.36	6.15 ± 1.36	6.23 ± 1.37	0.793^†^
Median [Q1–Q3]	6 [5–7]	6 [5–7]	6 [5–7]	
**Disaster preparedness perception scale score (28–140)**				
Mean ± SD	101.5 ± 13.6	103.6 ± 15.5	98.7 ± 9.8	**0.013** ^†^
Median [Q1–Q3]	101 [93–109]	104 [94–112]	99 [93–103]	

SD, standard deviation; Q1, 25th percentile; Q3, 75th percentile

^†^ Mann–Whitney U test

Table 3 summarizes the bivariate associations between disaster preparedness perception level and examined factors among final-year medical students. Higher disaster preparedness perception was significantly associated with older age (p = 0.010), male sex (p = 0.003), studying in Hatay (p = 0.002), previous participation in disaster-related health responses (p = 0.003), willingness to volunteer in disaster healthcare services (p < 0.001), and higher earthquake healthcare knowledge test scores (p = 0.002).

**Table 3:** Comparison of disaster preparedness perception levels by participants’ characteristics.

Variable	Disaster preparedness perception level				*p* ^‡^
	Low (≤ 101)		High (≥ 102)		
	n	%	n	%	
**Age group**					
≤ 24	63	62.4	38	37.6	**0.010**
≥ 25	38	43.7	49	56.3	
**Sex**					
Male	44	43.6	57	56.4	**0.003**
Female	57	65.5	30	34.5	
**University location**					
Hatay	48	44.0	61	56.0	**0.002**
Ordu	53	67.1	26	32.9	
**Previous participation in disaster-related health response**					
Yes	14	33.3	28	66.7	**0.003**
No	87	59.6	59	40.4	
**Willingness to participate in disaster healthcare services**					
Voluntarily	48	42.1	66	57.9	**<0.001**
Only if assigned / Unwilling	53	71.6	21	28.4	
**Earthquake healthcare knowledge test score**					
≤ 6	72	62.6	43	37.4	**0.002**
≥ 7	29	39.7	44	60.3	

Low (≤101) and high (≥102) disaster preparedness perception categories were defined based on the median HASPAHA score.

^‡^ Pearson’s chi-square test

Table 4 shows the results of the univariable and multivariable logistic regression analysis of factors associated with high disaster preparedness perception. In the univariable analyses, all six variables were significantly associated with high disaster preparedness perception. After multivariable adjustment, only male sex, studying in Hatay, willingness to participate voluntarily in disaster healthcare services, and an earthquake healthcare knowledge test score of ≥7 remained independently associated with high disaster preparedness perception. The multivariable logistic regression model demonstrated good fit (Hosmer–Lemeshow χ^2^ = 8.179, df = 8, p = 0.416), with a Nagelkerke R^2^ of 0.314. A total of 87 participants had high disaster preparedness perception (outcome events). No evidence of problematic multicollinearity was observed, with tolerance values ranging from 0.838 to 0.994 and VIF values ranging from 1.006 to 1.193.

**Table 4:** Univariable and multivariable logistic regression analysis of factors associated with high disaster preparedness perception.

Variable	cOR (95% CI)	*p*	aOR (95% CI)	*p*
**Age group**				
≤ 24	Ref		Ref	
≥ 25	2.14 (1.19–3.84)	**0.011**	1.71 (0.88–3.35)	0.116
**Sex**				
Female	Ref		Ref	
Male	2.46 (1.36–4.45)	**0.003**	3.13 (1.56–6.27)	**0.001**
**University location**				
Ordu	Ref		Ref	
Hatay	2.59 (1.42–4.73)	**0.002**	2.11 (1.04–4.29)	**0.039**
**Previous participation in disaster-related health response**				
No	Ref		Ref	
Yes	2.95 (1.43–6.07)	**0.003**	1.47 (0.63–3.41)	0.375
**Willingness to participate in disaster healthcare services**				
Only if assigned / Unwilling	Ref		Ref	
Voluntarily	3.47 (1.85–6.50)	**<0.001**	3.99 (1.94–8.20)	**<0.001**
**Earthquake healthcare knowledge test score**				
≤ 6	Ref		Ref	
≥ 7	2.54 (1.39–4.64)	**0.002**	2.95 (1.49–5.85)	**0.002**

cOR, crude odds ratio; aOR, adjusted odds ratio; CI, confidence interval; Ref, reference category

The multivariable logistic regression model had a Nagelkerke R^2^ of 0.314

In the additional multivariable logistic regression models including interaction terms, no statistically significant interactions were observed between university location and earthquake healthcare knowledge (aOR = 2.17, 95% CI: 0.54–8.70, p = 0.276) or willingness to volunteer (aOR = 3.09, 95% CI: 0.76–12.65, p = 0.117).

Sensitivity analysis using multiple linear regression with continuous score variables yielded findings consistent with the primary analyses, with male sex (B = 7.384, 95% CI: 3.775–10.993), studying in Hatay (B = 4.080, 95% CI: 0.183–7.977), willingness to volunteer in disaster healthcare services (B = 7.683, 95% CI: 3.973–11.394), and higher earthquake healthcare knowledge (B = 1.865, 95% CI: 0.547–3.183) remaining significantly associated with higher preparedness perception scores. The model had an R^2^ of 0.214 (S1 Table).

## Discussion

The present study suggests that disaster preparedness perception among final-year medical students cannot be interpreted solely as a reflection of disaster-related theoretical knowledge. Although earthquake healthcare knowledge scores were similar between students in Hatay and Ordu, preparedness perception scores were significantly higher among students in Hatay. This contrast suggests that factors beyond measured knowledge may be related to perceived preparedness, including contextual, motivational, and experiential influences. In addition, preparedness perception was independently associated with knowledge level, willingness to volunteer, male sex, and studying in Hatay in the multivariable analysis. Taken together, these findings highlight that perceived preparedness is a multidimensional construct that may reflect both knowledge-related and contextual factors. This distinction has important implications for medical education, suggesting that factors beyond theoretical knowledge may also be relevant to students’ perceptions of preparedness for disaster response.

There are limited studies in the literature assessing disaster preparedness perceptions among medical students. In a study conducted at a single medical faculty in Türkiye among fourth-, fifth-, and sixth-year medical students using the same scale, the mean preparedness perception score was 94.0 ± 13.7 overall and 101.3 ± 13.4 among sixth-year students [18]. The sixth-year mean score reported in that study fell between the values observed in Hatay and Ordu. These findings suggest that disaster preparedness perception levels among senior medical students in Türkiye fall within a broadly similar range when assessed using the same measurement instrument, although variation between institutions and study settings is evident. In contrast, studies conducted in Türkiye among Emergency Call Center employees and National Medical Rescue Team (UMKE) personnel [19], as well as healthcare personnel working in 112 Emergency Health Services Stations [20], using the same scale reported substantially higher preparedness perception scores (115.4 ± 16.7 and 118.0 ± 13.8, respectively) than those observed among medical students. These differences may reflect differences in study populations, professional experience, disaster-related training and routine exposure to emergency situations. Compared with medical students, emergency response personnel operate within clearly defined professional roles and are more likely to encounter real-world emergency and disaster scenarios. Therefore, higher preparedness perception among these groups may reflect the combined influence of multiple professional and contextual factors.

Studies conducted in different countries have examined disaster preparedness perceptions among medical students using various instruments, and have generally reported inadequate levels of perceived preparedness [12,21,22]. These studies suggest that medical students often report limited confidence in their ability to effectively respond to disaster situations. This issue is particularly relevant in disaster-prone countries, where near-graduation medical students represent a critical group of future physicians who may be deployed to frontline emergency health services in the event of large-scale disasters.

In the present study, Hatay and Ordu represent two distinct disaster exposure contexts within a broadly similar national medical education framework. Students studying in Hatay demonstrated significantly higher disaster preparedness perception compared with those in Ordu, and studying in Hatay remained independently associated with higher preparedness perception in multivariable analysis. Notably, knowledge test scores were comparable between the two groups, suggesting that the observed difference may not be explained solely by theoretical knowledge. The higher preparedness perception observed in Hatay may reflect students’ direct experience of a large-scale disaster. However, alternative explanations should also be considered. Although both universities operate within the same national medical education framework, unmeasured institutional or regional differences may partly explain the observed variation. Therefore, university location in this study should be interpreted as a proxy for a combination of disaster exposure context and unmeasured institutional and regional factors. Accordingly, the observed association with studying in Hatay may reflect not only disaster exposure but also other unmeasured differences between the two settings. In addition, because the study assessed perceived preparedness rather than actual disaster response performance, the higher scores observed in Hatay may reflect differences in awareness and self-appraisal following disaster exposure. Furthermore, selection bias related to the exclusion of some vulnerable students in Hatay cannot be ruled out. Nevertheless, previous research has reported an association between disaster exposure and higher preparedness perception among medical students [18].

In our study, willingness to volunteer in disaster healthcare services emerged as the strongest independent factor associated with higher preparedness perception. Similarly, a previous study conducted among medical students in Türkiye found that those willing to volunteer in case of disaster had significantly higher preparedness perception scores [18]. Similar associations have also been reported among other health-related student populations, where volunteering was positively associated with disaster preparedness levels [15]. However, not all studies demonstrate this relationship; for example, research conducted among nurses did not identify a significant association between preparedness perception and volunteering [23]. The association observed in our study may reflect the role of intrinsic motivation and professional responsibility in relation to perceived preparedness. Students who are more willing to assume responsibility in disaster settings may also report greater self-efficacy regarding their ability to function effectively in such contexts. Previous research has suggested that individuals with higher self-efficacy and confidence in their response ability are more likely to express willingness to volunteer [24]. However, because of the cross-sectional design of the present study, the causal direction of the relationship between preparedness perception and willingness to volunteer cannot be determined.

Students from Hatay and Ordu demonstrated similar earthquake healthcare knowledge test scores. However, when the total sample was examined, students with a knowledge score of ≥7 had nearly three times higher odds of reporting high disaster preparedness perception. This finding is consistent with previous studies that have reported a positive association between disaster-related knowledge and preparedness perception [3,25,26]. Higher levels of knowledge may be associated with greater confidence and self-efficacy in disaster response situations, which may be reflected in higher perceived preparedness. A greater understanding of disaster-related healthcare principles may be associated with students’ perceived ability to function effectively in disaster settings. These findings suggest that cognitive components of disaster education appear to be relevant to preparedness perceptions among future physicians. However, the similarity of earthquake healthcare knowledge scores between students in Hatay and Ordu despite significant differences in preparedness perception suggests that knowledge alone may not fully explain perceived preparedness. These findings suggest that disaster education may benefit not only from theoretical instruction but also from experiential and practice-based learning opportunities.

In the present study, male sex was independently associated with high disaster preparedness perception in multivariable analysis. The relationship between sex and disaster preparedness has been inconsistently reported in the literature. Several studies conducted among medical students and healthcare professionals using the same scale did not identify a statistically significant association with sex, although male participants generally reported higher preparedness scores [18,20]. Evidence from population-based studies has similarly suggested that men may report higher preparedness-related indicators [27,28]. However, findings across studies remain inconsistent, and the mechanisms underlying potential sex differences in preparedness perception are not fully understood. Therefore, the observed association should be interpreted cautiously.

Age and previous participation in disaster-related health response were significant in univariable analyses but were no longer significant after multivariable adjustment. This suggests that their apparent associations with preparedness perception may have been partly explained by potential confounding from other variables included in the model. Therefore, age and previous participation did not appear to have independent associations with preparedness perception after accounting for these covariates.

Taken together, the findings of this study suggest that disaster preparedness perception among final-year medical students is associated with multiple factors. While disaster-related knowledge was associated with preparedness perception, the similarity of knowledge scores between Hatay and Ordu indicates that knowledge alone may not fully explain perceived preparedness. Contextual factors related to disaster exposure and motivational factors reflected by willingness to volunteer may also be relevant. These findings highlight the multifaceted nature of perceived preparedness and suggest that disaster education may benefit from approaches that address not only knowledge acquisition but also experiential and motivational dimensions of preparedness.

This study has several limitations that should be considered when interpreting the findings. First, the cross-sectional nature of the study limits causal inference. Second, the use of self-reported data may have introduced recall bias and social desirability bias. Third, although a census-based recruitment approach was used, selection bias may have occurred due to differing response rates between the two universities and the ethical exclusion of some students in Hatay with earthquake-related bereavement or psychological vulnerability. These factors may have affected representativeness and may have led to overestimation of disaster preparedness perception in the Hatay group if non-participating or excluded students differed systematically from participants. In addition, no data were available for non-responders, precluding formal assessment of non-response bias. Furthermore, the inclusion of final-year medical students from only two institutions limits the generalizability of the findings to students in other year levels, graduating cohorts, universities, and regions, as well as to other healthcare professions and disaster-response settings. As the study was conducted during the early post-disaster period, the findings may also not be generalizable to later post-disaster periods. Because the comparison was conducted between two universities located in provinces with different disaster exposure profiles, residual confounding related to unmeasured institutional, regional, or contextual differences cannot be fully excluded. Fourth, the earthquake healthcare knowledge test focused specifically on earthquake-related topics, reflecting Türkiye’s predominant disaster type and targeting a core component of disaster preparedness relevant to the national context. However, while this disaster-specific focus is conceptually appropriate, the disaster preparedness perception scale assesses broader disaster preparedness. This difference in measurement scope should be considered when interpreting the association between knowledge and perceived preparedness. In the absence of a standardized or officially validated earthquake-specific healthcare knowledge assessment tool, the knowledge test was developed by the research team based on a national guide and reviewed by subject-matter experts. Nevertheless, comprehensive psychometric validation was not conducted; therefore, some degree of measurement bias should be considered when interpreting the knowledge assessment findings. In addition, dichotomization of continuous scores using median values may have reduced variability and statistical precision. However, supplementary sensitivity analysis using continuous variables yielded findings consistent with the primary analyses. Additionally, although some predictor categories included relatively few participants, the relatively small overall sample and limited number of outcome events may have reduced the precision and stability of some regression estimates and should be considered when interpreting the findings. Finally, the study assessed perceived preparedness rather than real-world performance, which may not fully reflect actual disaster response capacity. Despite these limitations, the study has noteworthy strengths. First, it was conducted shortly after the February 2023 Türkiye earthquakes, providing an opportunity to assess disaster preparedness perceptions in a post-disaster context. Second, the comparative design involving final-year medical students from two regions with different disaster exposure profiles allowed exploration of how disaster context may relate to preparedness perception within a broadly similar medical education system. In addition, focusing on final-year medical students—who will soon enter the healthcare workforce—provides insights into a critical and understudied group. The use of a validated disaster preparedness perception scale (HASPAHA) and multivariable analysis further strengthened the study.

## Conclusions

In conclusion, this study demonstrated that disaster preparedness perception among final-year medical students was associated with both individual and contextual factors. Male sex, studying in Hatay, willingness to volunteer in disaster healthcare services, and higher earthquake-related healthcare knowledge were independently associated with higher perceived preparedness. These findings suggest that beyond theoretical knowledge, regional disaster exposure context and motivational factors may also be relevant to preparedness perceptions among future physicians. Accordingly, undergraduate medical curricula could consider combining structured disaster preparedness education with experiential and practice-oriented training opportunities, particularly in disaster-prone settings. In addition, providing opportunities for voluntary participation in disaster-related activities while fostering students’ motivation to engage in disaster response may be relevant for promoting preparedness perceptions. Future research should employ longitudinal designs and include objective measures of disaster response competence to better understand how perceived preparedness translates into actual performance in real disaster settings.

## Supporting information

S1 Table Multiple linear regression sensitivity analysis of factors associated with continuous disaster preparedness perception scores. (DOCX)

S1 File Data. (XLSX)
