# Supplementary material for: Disaster preparedness perceptions and associated factors among final-year medical students in Hatay and Ordu, Türkiye
Source: PLoS One. 2026 Oct 6;21(10):e0358641. doi: 10.1371/journal.pone.0358641 (PMC13641344; doi:10.1371/journal.pone.0358641)
Supplement: Supplementary file 1 [file pone.0358641.s001.docx]

**S1 Table. Multiple linear regression sensitivity analysis of factors associated with continuous disaster preparedness perception scores.**

| **Variable** | **B** | **95% CI for B** | | **Std. β** | ***p*** |
| --- | --- | --- | --- | --- | --- |
| Age (continuous) | -0.244 | -1.438 | +0.949 | -0.028 | 0.687 |
| Male sex (vs female) | +7.384 | +3.775 | +10.993 | +0.271 | **<0.001** |
| Studying in Hatay (vs Ordu) | +4.080 | +0.183 | +7.977 | +0.148 | **0.040** |
| Previous participation in disaster response (vs none) | +1.450 | -3.173 | +6.072 | +0.044 | 0.537 |
| Willingness to volunteer (vs assigned/unwilling) | +7.683 | +3.973 | +11.394 | +0.276 | **<0.001** |
| Earthquake healthcare knowledge score (continuous) | +1.865 | +0.547 | +3.183 | +0.186 | **0.006** |

Overall model p < 0.001 (ANOVA F-test). All variance inflation factor (VIF) values were < 5.
